# Utility of learning plans in general practice vocational training: a mixed-methods national study of registrar, supervisor, and educator perspectives

**DOI:** 10.1186/s12909-016-0736-8

**Published:** 2016-08-19

**Authors:** Belinda Garth, Catherine Kirby, Peter Silberberg, James Brown

**Affiliations:** 1 Southern General Practice Training (Eastern Victoria GP Training), 50 Northways Road, Churchill, Victoria, 3842 Australia; 2 North Coast General Practice Training, Ballina, NSW Australia; 3 School of Rural Health, Faculty of Medicine, Nursing and Health Sciences, Monash University, Victoria, Australia

**Keywords:** Learning plans, Learning planning, Qualitative, General practice training, Deliberative learning, Socio-cultural theory, Socio-material theory, Adult learning theory

## Abstract

**Background:**

Learning plans are a compulsory component of the training and assessment requirements of general practice (GP) registrars in Australia. There is a small but growing number of studies reporting that learning plans are not well accepted or utilised in general practice training. There is a lack of research examining this apparent contradiction. The aim of this study was to examine use and perceived utility of formal learning plans in GP vocational training.

**Methods:**

This mixed-method Australian national research project utilised online learning plan usage data from 208 GP registrars and semi-structured focus groups and telephone interviews with 35 GP registrars, 12 recently fellowed GPs, 16 supervisors and 17 medical educators across three Regional Training Providers (RTPs). Qualitative data were analysed thematically using template analysis.

**Results:**

Learning plans were used mostly as a log of activities rather than as a planning tool. Most learning needs were entered and ticked off as complete on the same day. Learning plans were perceived as having little value for registrars in their journey to becoming a competent GP, and as a bureaucratic hurdle serving as a distraction rather than an aid to learning. The process of learning planning was valued more so than the documentation of learning planning.

**Conclusions:**

This study provides creditable evidence that mandated learning plans are broadly considered by users to be a bureaucratic impediment with little value as a learning tool. It is more important to support registrars in planning their learning than to enforce documentation of this process in a learning plan. If learning planning is to be an assessed competence, methods of assessment other than the submission of a formal learning plan should be explored.

**Electronic supplementary material:**

The online version of this article (doi:10.1186/s12909-016-0736-8) contains supplementary material, which is available to authorized users.

## Background

Over the past three decades, adult learning theory has been a popular model among educators. This model places responsibility on learners to diagnose their own learning needs, design their learning experiences, locate resources and evaluate their progress [1]. Learning planning is the deliberative process a learner engages in to identify their learning needs, set time aside to acquire new knowledge or skill, and undertake activities which move them toward attaining their goal [2]. Learning plans are a tool intended to assist learners to cohesively and strategically identify, plan and document their learning needs and activities; as well as to prompt reflection of their learning experiences and progress [3, 4]. Learning plans and portfolios were introduced to promote a self-directed learning process and enable individuals to monitor their progress [5]. Subsequently, the submission of a learning plan has become a common assessment requirement as evidence of both learning planning and of active engagement in learning activities. Internationally, learning plans and portfolios are a familiar activity for those engaged in general practice (GP) training, forming part of the credentialing process.

While the importance of planning one’s learning is well documented, there is a small but growing number of studies reporting that documentation of planning in formal learning plans is not well accepted or utilised among adult learners in general [6–8] and in the context of medical education [9–12]. There is currently a lack of research that examines this apparent contradiction. We chose to examine this issue within the Australian context because formal learning plans are an integral and mandatory component of GP vocational training and yet there is doubt about the effectiveness of their use.

### The current study

This study examined the use and perceived utility of formal learning plans in Australian GP vocational training, and was guided by three overarching research questions:

1. How do Australian General Practice Training (AGPT) registrars identify and address their learning needs?
2. What is the perceived utility and acceptance of Regional Training Provider (RTP) formal learning plans?
3. How might registrar learning planning be supported effectively?

A large national research project was conducted, using a mixed-method design that included: analysis of online learning plan usage data; focus groups and interviews with GP registrars, supervisors and medical educators (MEs); self-report log of registrar learning activities; and a Delphi process to establish a best practice consensus statement for learning plan use.

This paper reports findings related to: registrar use of formal learning plans, supervisor role in registrar learning plans, and perceptions of formal learning plans.

## Methods

This research endeavor was a collaboration by three of the 17 Australian GP vocational training RTPs in 2015. Whilst learning plan use is mandated by the two Australian colleges of general practice and registrars are required to submit a documented learning plan for completion of training, there are no specific guidelines on the required content, format, and delivery of completed learning plans. RTPs across Australia have typically worked independently to develop and deliver a learning plan structure for their registrars to use. As may be expected, there is considerable variation in the learning plan tools and approaches to learning planning across RTPs.

The three RTPs in this investigation engaged in this collaboration because of a mutual interest in examining the utility of learning plans. Between these three RTPs there was a broad representation of registrar training and of approaches to learning planning. Collectively, they were responsible for the training of 505 registrars across a wide range of environments from urban to very remote serving both economically advantaged and economically very disadvantaged communities with indigenous and non-indigenous populations. Their registrar cohorts were from both Australian and international backgrounds. Each RTP had independently developed their own version of a formal learning plan and had different approaches to induction and support for registrars in their use of learning plans. RTP 1 had recently transitioned from an electronic, static document to an interactive online learning plan, RTP 2 used a traditional paper-based learning plan, and RTP 3 used an online learning plan. These are further outlined in Additional file 1. Each RTP discussed the requirement of completing a learning plan at orientation. RTP 1 and RTP 3 both emphasised the mandatory status of documented learning plans and followed them up if not submitted. RTP 2 was less formal in their requirements for the submission of documented learning plans.

Study participants were drawn from these three RTPs. Data were collected through electronic learning plan usage data (available from RTP 1 and 3), separate focus group discussions with registrars, GP supervisors, and MEs, and telephone interviews with GPs who had recently completed training.

### Sample and recruitment

Sampling was purposive to explore the perspectives of intended users of learning plans (i.e. registrars from different stages of training) and those tasked with responsibility to ensure learning plan completion (i.e. GP supervisors and MEs). Participants included 18 registrars in their first year of training (GPT1&2), 17 registrars in their second year of training (GPT3&4), 16 GP supervisors, 17 MEs and 12 GPs who had completed their training within the past 12 months.

Registrars were recruited through email invitations sent by the administration staff and by face-to-face invitations at RTP training workshops. Participation was voluntary. Registrars who expressed an interest in participation were provided with an explanatory statement and consent form. Those who consented to participate were allocated to a focus group. Supervisors and MEs were recruited through email invitations sent by administrative staff and by the medical educators in the research team. Those interested in participating were asked to contact the researchers (CK and BG) who provided more information and scheduled the focus groups. Where possible, all focus groups were scheduled around RTP and other relevant GP educational events, to facilitate recruitment and make participation easier. All participants were offered retail gift cards as a reimbursement for their time.

GPs who had recently completed their training were recruited through email invitations from administrative staff and sent an explanatory statement and consent form if interested. Individual telephone interviews were scheduled for those consenting to participate.

### Data collection

Learning plan usage data was collected retrospectively for a 6 month period (semester two 2014), using de-identified group data extracted from the online learning plan tools used at RTP 1 and 3. No usage data was available from RTP 2. These data were collected to gain an objective indication of whether and how registrars were using their electronic learning plans.

Twelve focus groups (Table 1) were conducted with AGPT registrars, GP supervisors and MEs from each of the RTPs. Focus groups were chosen as a method of data collection to obtain detailed information from a range of participants simultaneously. Interaction between participants encouraged clarification of ideas. Twelve one-on-one telephone interviews (RTP 1, *n* = 2; RTP 2, *n* = 5; RTP 3, *n* = 5) were conducted with GPs who had recently completed training as these participants were more geographically disparate. All data were collected between October 2014 and February 2015.

**Table 1:** Focus group participant demographics for each Regional Training Provider (RTP)

	Participants (*n*)	Focus group duration (min)	Gender		Age range (mean)	Medical graduate status			Years as a GP, range (mean)	Years as a GPS/ME, range (mean)
RTP 1			Male (*n*)	Female (*n*)		AMG^ (*n*)	IMG^^ (*n*)	Not answered		
Registrars, GPT1&2^a^	5	73	3	2	30–47 (36.2)	2	3	–	–	–
Registrars, GPT3&4^b^	5	58	1	4	29–57 (38.6)	4	1	–	–	–
GP Supervisors (GPS)	4	59	2	2	48–59 (53.5)	4	0	–	20–30 (25.8)	8–27 (14.5)
Medical Educators (MEs)	5	65	0	5	31–55 (39.4)	4	1	–	2–25 (9.2)	0.5–15 (5.9)
RTP2										
Registrars, GPT1&2^a^	5	44	3	2	28–34 (31.6)	3	2	–	–	–
Registrars, GPT3&4^b^	5	64	2	3	29–47 (34)	2	3	–	–	–
GP Supervisors (GPS)	5	67	3	2	47–62 (55.4)	3	1	1	11–34 (23.2)	3–29 (17.4)
Medical Educators (MEs)	5	76	1	4	39–67 (56.6)	4	1	–	14–42 (31.4)	4–20 (12.4)
RTP3										
Registrars, GPT1&2^a^	8	70	4	4	28–39 (32.3)	8	0	–	–	–
Registrars, GPT3&4^b^	7	61	3	4	28–45 (33.7)	4	3	–	–	–
GP Supervisors (GPS)	7	58	5	2	39–62 (52.4)	4	3	–	13–35 (23)	3–27 (11.9)
Medical Educators (MEs)	7	59	5	2	35–70 (51)	4	3	–	4–41 (20.3)	3–16 (9)
Total	*n* = 68	x = 63	*n* = 32	*n* = 36	–	*n* = 46	*n* = 21	*n* = 1	–	–

^a^GPT1&2 = registrars in their first year of training

^b^GPT3&4 = registrars in their second year of training

^*AMG* Australian Medical Graduate

^^*IMG* International Medical Graduate

Interviews and focus group discussions were facilitated by experienced qualitative researchers, CK and BG, who did not know the participants. JB co-facilitated five of the focus groups with either BG or CK at RTP 1 and RTP 2 which were not the RTPs in which he worked. While JB was not known to the registrars in these groups he was acquainted with some of the supervisors and MEs.

Interviews and focus groups utilised a semi-structured format with a schedule informed by the literature and expertise of the research team (see Additional file 2). The schedule was modified in light of experience of the initial focus groups and interviews. The semi-structured format allowed for focused enquiry and naturalistic discussion.

### Data analysis

Learning plan usage data were exported from the online learning plan software and analysed using Statistical Package for the Social Sciences (SPSS) version 22.

Focus groups and interviews were digitally audio-recorded, transcribed verbatim, and identifying information removed. Individual voices were not identified in the focus groups. Qualitative data analysis software NVivo version 10 was used to manage the data.

Template analysis [13] was used to identify key themes and relationships from qualitative data. An initial template was generated by the authors who independently analysed the same three focus group transcripts. We used both deductive coding guided by the research questions and inductive open coding to allow for emergent themes. Codes were grouped into thematic categories. The investigators met to compare coding and establish an agreed initial template for coding the remainder of the transcripts. The template was refined through subsequent coding of the remaining transcripts by BG. The final template consisted of four levels of coding (Table 2). To ensure validity and reliability of interpretations, emerging themes and codes were presented to the other three investigators for comment throughout analysis to enable cross-checking and to achieve consensus on the final template.

**Table 2:** Final template^a^

1. Identifying learning needs a. Drivers of learning b. Barriers to identifying registrar learning needs (gaps in knowledge & skill) c. Learning needs missed by registrars 2. Addressing learning needs and learning planning a. Role of registrars b. Role of supervisors c. Role of medical educators d. Role of training providers **3. Formal learning plans** ** a. Perceptions of learning plans** ** i. Negative** ** 1. Bureaucratic exercise** ** 2. Unsuitable for some learning styles** ** 3. Questioning the need for a learning plan** ** 4. Lack of buy-in** ** 5. Unsuitable for adult learners** ** 6. Does not work in practice** ** 7. Exposing registrar weaknesses** ** ii. Positive** ** 1. Good way to store, document, or reflect on learning needs** ** 2. Brings focus to learning** ** 3. Value of learning plans not being appreciated until later** ** 4. Promotes learner reflection** ** 5. Helpful for remediation** ** 6. Encourages independent thinking and self-directed learning** ** 7. Good idea at a macro level** b. Supervisor & ME perceptions - how learning plans are supposed to be used ** c. Registrar use of RTP formal learning planner** ** i. As intended by RTP** ** ii. To meet requirement but did not find it helpful** ** iii. Under-used the learning plan** ** iv. Created their own learning plan (uncommon)** d. Facilitators to learning planner use e. Barriers to learning planner use 4. Suggestions for improvement		

^a^Third and fourth level codes from categories 1–2, 4 have not been included here for brevity. Category 3 (in bold) is presented in this paper

A number of theoretical lenses informed our initial discussions and analysis. This reflected the eclectic research backgrounds of the research team and the range of perspectives that our data provided. These lenses included: Knowles’ adult learning theory [14, 15], Billet’s theory of interdependence between social and individual agency [16], Bandura’s social-cognitive theory [17], Wenger’s socio-cultural theory [18] and socio-material theory outlined by Fenwick and Edwards [19].

In our early discussions and analysis we recognised the limitations of adult learning theory; the tenet that learners direct their learning was helpful but did not always fit. The context of registrar learning went beyond an individualistic experience, to a hybrid of individual and social influences (i.e. patients, other registrars, supervisors and MEs) and also included the use of tools (i.e. computers, paper notebooks). Recognising learning as being the concern of not just the learner but also of the communities in which they learn is a tenet of socio-cultural theories, which we found useful for examining our data. Additionally, with our focus on the perceived utility and acceptance of formal learning plans, we found a socio-material perspective particularly useful in understanding the role and place of learning plans and other material artefacts in registrars’ learning [19].

Three forms of triangulation were used in this study; data source triangulation (across participants), data type (qualitative and quantitative) and researcher triangulation (across investigators in the collection and analysis processes) [20, 21]. By sourcing participants from a range of learning plan user groups we were able to gain a broad understanding of the experience of learning planning and formal learning plans in GP vocational training. By using qualitative interview and focus group data and quantitative analysis of electronic learning planner use, we were able to compare features of what participants said they did against what they actually did. By engaging investigators from differing backgrounds for data collection and analysis we strengthened the reliability and validity of our data and its analysis [20, 21]. The multiple sources of triangulation used in this study increase the credibility of the findings. Additionally, analysis of focus group data included examination of negative cases (disagreement in the group) [22] thus increasing the rigor of our investigation.

## Results

1. Registrar use of formal learning plans
  Quantitative data
    RTP 1
      Of the 80 registrars at RTP 1, only 34 (42.5 %) submitted their learning plan (Table 3). Chi square tests of independence were performed to examine whether there was any significant correlation between registrar characteristics and their completion of a learning plan. As displayed in Table 3, registrars who completed their medical degree overseas (IMG) were more likely to complete a learning plan than those who studied locally (AMG), χ (1) = 4.351, *p* = .037. There was no significant relationship between learning plan completion and gender or year of training commencement *p* > .05.

**Table 3:** Breakdown of learning plan completion by registrar gender, cohort, and medical graduate status

Characteristic	Learning plan submitted RTP 1 *n* (%)		Learning plan used RTP 3 *n* (%)	
	Yes	No	Yes	No
Gender				
Female Male	25 (47 %) 9 (33 %)	28 (53 %) 18 (77 %)	62 (69.7 %) 29 (74.4 %)	27 (30.3 %) 10 (25.6 %)
Cohort				
2008 2009 2010 2011 2012 2013 2014	0 (0 %) 0 (0 %) 2 (67 %) 3 (60 %) 9 (56 %) 9 (35 %) 11 (39 %)	1 (100 %) 1 (100 %) 1 (33 %) 2 (40 %) 7 (44 %) 17 (65 %) 17 (61 %)	0 (0 %) 1 (100 %) 0 (0 %) 3 (42.9 %) 24 (75.0 %) 36 (70.6 %) 27 (77.1 %)	1 (100 %) 0 (0 %) 1 (100 %) 4 (57.1 %) 8 (25.0 %) 15 (29.4 %) 8 (22.9 %)
Medical graduate status*				
AMG IMG NZMG	22 (36 %) 12 (63 %) 0 (0 %)	39 (64 %) 7 (37 %) 0 (0 %)	45 (64.3 %) 44 (78.6 %) 2 (100.0 %)	25 (35.7 %) 12 (21.4 %) 0 (0 %)

*Abbreviations*: *AMG* Australian Medical Graduate, *IMG* International Medical Graduate, *NZMG* New Zealand Medical Graduate

*p < 0.05 for RTP 1
    RTP 2
      Usage data were not available for RTP 2, so an attempt was made to estimate usage by examining end-of-training interview data relating to learning plan use and utility. Of the 38 registrar exit interviews undertaken in 2014 and the first half of 2015, the majority of registrars indicated that they did not use the learning planner, and did not find it useful (see Additional file 3).
    RTP 3
      Data were collected for 128 registrars at RTP 3. Data extracted from RTP 3’s online learning plan provided information about registrars’ frequency and patterns of learning plan use, if they opted to use the online plan. Seventy one per cent of registrars (*n* = 90) entered one or more learning items (i.e. learning need or activity) on their plan, with an average of 13 learning items entered during the semester. Nine percent of registrars (*n* = 12) did not access the learning planner at all during the data collection period. There was no significant relationship between the learning planner use and registrar gender, year of training commencement, or medical graduate status.
      Data on the completion of learning items indicated that 82 % of items were entered and ticked off as completed within the same day by registrars. The method of learning plan item entry was also examined, to identify how registrars used the learning planner. The majority of learning items were entered directly into the planner by registrars (Table **4**).

**Table 4:** Method of learning plan item creation/entry

Method of item entry	*n* (%) of learning plan items entered
Directly by registrar	1384 (80.5 %)
Directly by supervisor	15 (0.9 %)
Auto-populated via learning planner tools (e.g. College curriculum, procedural skills log)	321 (18.6 %)
Total	1720 (100 %)
  Qualitative data
    Data from focus groups and interviews indicated that when planning their learning, registrars typically made informal notes of their learning needs as these needs arose. Most commonly this was to record a learning need in reaction to what had arisen from a patient consultation. These notes were either paper or electronic, or simple mental notes. These notes served as a reminder or prompt for following up later and did not include detail about ‘how’ they planned to address the identified learning need. Formal learning plans from all RTPs were reportedly used to a much lesser extent by registrars. Table 5 details the range of registrar reported approaches to learning plans.

**Table 5:** Reported use of formal learning plans-registrar perspective

Type of learning plan use	Reasons	Quotes
Registrar used learning plan as intended by RTP		
Actions: • Deliberative planning and goal setting (not just a log of learning activities). • Entered learning items into online learning planner. • Discussed learning plan with supervisor.	• Facilitated strategic planning. • Facilitated keeping track of learning and planning tasks for the weeks ahead. • Ensured outcomes were achieved (for some). • Email reminders helpful.	“I could plan and prioritise and it kind of helped because I was in a remote area … I could plan what courses and things I actually wanted to do to get those learning objectives done and that meant I could book leave.” (GP102)
Registrar used learning plan to meet requirement but did not find it helpful		
Actions: • Fabricated some parts of the learning plan. • Inputting ‘easy’ learning activities for the purpose of ticking them off. • Retrospective completion (rather than prospective planning).	• Was a requirement of training but not a meaningful activity for registrar. • Forgot about goals that were initially written down.	“… I had to find it on my computer where I’d put it … because I hadn’t seen it in 6 months, and then I’m sitting there thinking, ‘What did I write last time and what am I going to write this time? And I don’t want it to look the same’” (RTP1, GPT1&2 s)
Registrar under-used learning plan		
Actions: • Learning planning (process) was occurring but learning was not documented in a learning plan. • Use of learning plan intermittently. • Started but stopped using a learning plan.	• Lack of meaningful follow-up by the RTP or supervisor. • Lack of time. • Wasn’t a priority. • Didn’t find it useful. • Too complicated and overwhelming. • Forgot to use it. • Lost motivation.	“I’m a GP supervisor and a medical educator and it’s my impression that the vast majority of registrars don’t actively use learning plans.” (RTP3, Supervisors)
		“I wouldn’t get mine actually written till maybe halfway through or something like that, I’d usually have to be nagged a bit by [the RTP] to complete it.” (GP102)
Registrar created their own learning plan (uncommon)		
Actions: • Generating own format was reported to be more efficient, more useful, and reflective of their own learning style. • Tools used for learning planning: -notebook -post-it notes -spreadsheet -pre-existing online reminder system -Word document -GoogleDocs	• Convenience • Personal preference • More useful • More accessible	“I would just do it myself, make up my own spreadsheet and do it myself on the computer.” (GP102)
		“I just found that more useful because it was real in terms of what was coming in. And to…it was accessible. Whereas I didn’t actually find the real learning plan accessible. But that’s just my style of learning. But I feel like a lot of people feel that way.” (GP202)
2. Supervisor role in registrar formal learning plans
  The online learning plan at RTP 3 was designed to be accessible to registrars, supervisors and MEs; potentially facilitating discussion and collaborative identification of learning needs. The learning plan was intended to be actively read and ‘acknowledged’ regularly during a semester by a supervisor. However, supervisors accessed the learning plan on average only once per semester for a given registrar (Table 6).

**Table 6:** Access to learning planners for each registrar (Semester 2, 2014)

	Number of times LP was accessed	
LP access for each registrar	Range (min–max)	Mean (SD)
Access by registrar	0–83	12.93 (13.82)
Access by supervisor	0–15	1.16 (2.11)
Access by ME	0–9	1.59 (1.82)
  Qualitative data suggested some supervisors across the three RTPs did take an interest in registrar learning plans (e.g. using them as a focus for teaching sessions or making suggestions to registrars to improve their learning plan). However, the more common experience was infrequent supervisor engagement with their learning plans.
  “I would say they have minimal involvement in the actual learning plan … it’s not like they sat down with me and went through my learning plan and my learning goals.” (GP202)
  “… both supervisors said that learning planning was pointless, learning planners themselves were pointless, so neither of them ever looked at them”. (GP203)
  Registrars were more likely to use a learning plan if their supervisor was supportive and took an active interest in it.
  “I worked with one supervisor for a year who would prompt me … I think we had a half hour meeting every week, which was quite generous compared to some other places and it did get asked at every one whether I’d been using it and that sort of thing, you should be using it, you should be checking it, and I did tend to use [the learning planner] a lot more time, whereas I’ve been other places where it’s not mentioned and I sort of, it just slips off the radar …” (RTP3, GPT3&4 s)
3. Perceptions of formal learning plans
  Overall, seven categories each of positive and negative perceptions of formal learning plans were identified from the qualitative data (see Table 2). Whilst there were equal number of positive and negative categories, a much larger portion of the data were coded under negative categories; indicating participants had more to say about why they did not value learning plans. Most categories emerged across all participant groups, and are summarised below. Additional data from registrars and GPs, and from GP supervisors and MEs are presented separately in Tables 7 and 8 respectively.

**Table 7:** Registrar perceptions of learning plans

Category	Registrars and GPs (recently completed training)
Positive perceptions	
Good way to store, document, or reflect on learning needs	“Well I suppose it’s a way to direct our study but also, you know, you can reflect back on it at the end of term and go, ‘Oh, did I actually complete that goal?’ And, ‘Where did I fall down?’” (RTP1, GPT3&4 s) “I do like the concept of the learning plan because … if I didn’t write it down I wouldn’t remember it, so I really do, I really found it useful to write down what topics I needed to know more about. Because it would then give me something to focus on” (GP202)
Brings focus to learning	“… when I started using it in my GPT1 I found it really helpful … it actually keeps a track of my planning” (GP301) “I find that learning plan is a very helpful way as to help me to remember what sort of things I need to learn, what sort of things I want to know more” (GP302)
Value of learning plans not being appreciated until later	“… in the last year or so when I was kind of thinking this actually wasn’t a bad idea then I started to be more kind of pragmatic about it and thought it was actually kind of a useful thing” (GP102) “I am now on the learning planner bandwagon” (GP304)
Promotes learner reflection	“…for people like that, that have no idea or don’t have that cohesive or reflective way of learning, it’s good to have a learning planner there so they can then go back and reflect on what they’ve learnt … Because not everybody is alike in terms of being proactive with their learning” (GP304)
Encourages independent thinking and self-directed learning	“So I find my current learning plan so much more useful because it’s entirely directed by me and it’s focused on what I’m seeing every day” (RTP2, GPT3&4 s)
Good idea at a macro level	“I do like what it’s trying to achieve but I don’t know that it quite does it very effectively” (RTP1, GPT3&4 s) “… conceptually it was helpful … it helped to demonstrate what you might do if you were identifying learning deficits. But I don’t think as an actual tool I ever used it in a useful way. But the concept that it transmitted was helpful” (GP203)
Negative perceptions	
Bureaucratic exercise	“It was a bureaucratic process, more than facilitating learning” (GP101) “I only fill the learning planner in to satisfy the requirement that it’s filled in. It doesn’t actually aid my learning” (RTP3, GPT1&2 s) “It does seem like a bit of an administrative way of proving that we’re learning something … Not so much for our sake” (RTP2, GPT3&4 s) “Definitely is just an admin task … it’s like writing down if I ate breakfast … where does that benefit me apart from me having proof that I ate breakfast. If I studied for five hours and I spend 30 min documenting my study for five hours it’s a waste of a half-hour to me” (RTP3, GPT3&4 s)
Unsuitable for some learning styles	“Because I’m overseas graduate I’m not sure about the medical school assessment here, I’m not familiar with any log book, portfolio, but we only had the main exam as our [assessment], that’s what we target for, and I’m so I’m not familiar with these kind of assessments” (RTP1, GPT1&2 s) “… you’re sort of forcing your learning experience to conform to something that somebody else has kind of put upon you” (GP102) “it could be of benefit to RTPs to recognise and I’m sure they do recognise that there are different learning styles, so tying registrars to a compulsory style of learning doesn’t work for everybody” (GP204)
Questioning the need for a learning plan	“I don’t like doing the learning planner because it’s a waste of my time to try and dig out what I need to know, put it onto the system and then learn from that” (RTP3, GPT1&2 s) “I personally feel like we just do it, you know, just as something we need to complete” (RTP1, GPT1&2 s) “The learning planner really doesn’t inform them of anything because I could just make up a list and tick it off without looking it up, and I don’t see the function” (RTP3, GPT1&2 s)
Lack of buy-in	“… if you want me to do something give me a good reason why” (RTP2, GPT3&4 s) “So I think for the most part it’s supposed to be something that enables you … To learn or to direct you really if you like, or give you a direction before you start, you know? But I find for most of it, it just ends up being one of those barriers where you say, ‘Okay, it’s something I’ve got to do to get over that, sort of hurdle …’ … Just do it. And so it doesn’t have that impact I think that it’s intended for, and I think most people just write what they need to get past with it” (RTP1, GPT1&2 s)
Unsuitable for adult learners	“I feel like it’s an insult to our intelligence; like you’ve gone through bloody 20 years of study plus and you know, we know how to learn, we’re not dumb” (RTP3, GPT1&2 s) “I’m a mature aged student … and I think that learning plan—I don’t know, it made us feel real ‘schoolish’” (RTP2, GPT3&4 s) “It doesn’t make the slightest bit of sense because either you’re a grown up learning something or you need to be asked how you feel about learning something; they are completely separate populations, so if you’ve got adult learning—if you’ve got adults you don’t need to do all of that. [group agreement]” (RTP2, GPT3&4 s) “I’ve learnt how to organise my learning and my overall feedback to my training provider is, please just respect me and please don’t insult me as an adult learner” (GP101) “But it’s the sort of thing where as an adult learner I can, I can plan my learning and I’ve decided this method of learning is not, is not valuable to me so I’m not going to engage in it thank you very much” (GP101) “I think people feel a little bit babied by the whole experience” (GP203)
Does not work in practice	“Like I’ve got a plan in my head of what I’m going to do, how I’m going to do it and if the learning planner happens to intersect at points in time then I will tick them off at that point, but I don’t let the learning planner guide me at all” (RTP3, GPT1&2 s) “… one of the questions on … their sample learning planner was, ‘how will I know when I’ve achieved that?’ And that’s a pretty difficult question to answer … you know, be able to do a good pap smear, well how do you know when you, when you’ve mastered that, like when the patient doesn’t jump up off the bed and run out flinging the speculum back at you?!” (GP102)
Exposing registrar weaknesses	“No one wants to write it down. No one wants to write down the weak spots.” (RTP3, GPT3&4 s)

**Table 8:** GP Supervisor and Medical Educator perceptions of learning plans

Category	GP Supervisors and Medical Educators
Positive perceptions	
Brings focus to learning	“that’s the best way of doing it because you’ve then got your awareness focused on, ‘Okay, what am I uncomfortable with? What am I not quite on top of? What might have changed? What haven’t I looked at for a while?’ And that’s how we stay safe and current” (RTP2, Supervisors) “I think in recording [learning needs] they commit to it a bit” (RTP2, MEs)
Promotes learner reflection	“Well that is reflection though, what have I learnt today? I think, we’ve been trying to ask registrars, particularly when we have complicated topics like wounds for instance. And we have at a workshop somebody who’s very passionate about wounds and come and speak … and we don’t take much away from it, unless we write down stuff, so we’ve started to ask people to identify, you know, 3 or 4 things that they can take away and reflect on and write it down and use their learning plan as log for that, because otherwise there’s a lot of information and yeah if their heads like mine it just goes. So I think reflection can happen with learning logs as well as learning plans” (RTP3, MEs)
Helpful for remediation	“I have also been involved in remediating a number of registrars who’ve failed exams and when you actually make them produce a learning plan that addresses the active deficiencies that are identified, they actually improve their performance so it’s a useful tool” (RTP3, Supervisors)
Encourages independent thinking and self-directed learning	“So the idea is, that this is trying to, that’s the way I understand it, trying to get them to be thinking independently about what else do I need to do” (RTP3, MEs)
Negative perceptions	
Bureaucratic exercise	“they do actually have all that in their heads but they find it frustrating sort of being pushed to do it in a written down way I find” (RTP1, MEs)
Unsuitable for some learning styles	“I never planned my learning and I still don’t, and I did okay” (RTP2, MEs) “So I think people inherently do what works for them … it may not be a written, formal thing … it’s not useful for them write it down sometimes; it feels like a waste of their time to write it down” (RTP1, MEs) “I think it depends how people learn; I quite like them actually. I don’t think they should be mandatory but I think for some people they’re much more useful than others” (RTP1, MEs)
Questioning the need for a learning plan document	“I agree that what we’re talking about this sort of floating learning plan that you work out on the run and update verbally, is probably much more a useful thing” (RTP1, Supervisors) “It should be a verb not a noun, and the trouble is it’s talked about as a noun, but it’s actually a verb, it’s a process, it’s an action thing, but it gets talked about as a noun, like something that you can hold or look at. And that’s where it gets all mixed up because we get so focused on the noun and we don’t concentrate on the verb, which is how do you actually learn? And what’s your process for learning? That’s where the problem is” (RTP2, MEs) “The thing is people may well have a plan for their learning but they just don’t structure it in that way” (RTP1, MEs)
Lack of buy-in	“Some ivory tower educational thing, probably wasn’t in medicine, it was probably…It was someone in that educational ivory tower or sitting in Canberra [Australia’s Capital City] who decided that’s a good idea” (RTP3, Supervisors) “I don’t have a lot of evidence that compelled me as a supervisor to change my behaviour … I think that if I was more convinced that they were a useful thing rather than a tick box for getting through the training then that may be a helpful thing” (RTP1, Supervisors) “So we try to tow the party line without really believing in it ourselves” (RTP3, MEs)
Unsuitable for adult learners	“to get a … 30-year-old and sit down and say well this is how you’re going to learn, write down every night and tick it off and tick the box, is a little bit insulting for them” (RTP3, Supervisors) “I’m really not sure it’s necessary for experienced learners” (RTP3, Supervisors) “It’s just such a stupid concept for high achieving adults, which is what we’re dealing with, it’s just the most ridiculous idea.” (RTP2, MEs)
Does not work in practice	“There is a whole lot of just in time stuff that I think kind of can really go missing if everything turns into a formal kind of process” (RTP1, Supervisors) “… there’s no benefits to the registrar from doing the learning plans the way that we do it now … because of lack of time it’s for 6 months, and you’re new to a practice and you don’t really know what you want to learn about … most of the learning is, ‘I’ve seen this patient today,’ or like, ‘I heard about this thing and I don’t know much about it. How am I going to know about it a bit more?’ And that’s how most of the learning happens; it’s not you sitting down at the beginning of the term and then planning for the whole year, it’s just—that’s not what happens in real life” (RTP1, MEs)
Exposing registrar weaknesses	“Because they’re admitting there’s things that they don’t know and it’s down in hard copy, print, that ‘I don’t know this’ and it can—it’s one thing to identify a learning need in yourself but sometimes when you look at them and you think, ‘That’s really trivia, I really should have known that,’ or, ‘That’s important, I really should have known that,’ and do you really want that advertised that you had to look that up?” (RTP2, Supervisors) “… one registrar that was struggling and about his learning plan of what he felt was missing. Initially he actually felt embarrassed to talk about those learning plans cos he felt ‘well I shouldn’t tell anyone that I’m not good with managing UTI’. And he wouldn’t even tell me, he just said that ‘I’m just very afraid of that’” (RTP3, MEs)

  Positive perceptions
    Good way to store, document, or reflect on learning needs
      Some registrars and GPs perceived that learning plans were a good way to store learning needs. For some, the act of noting learning needs down meant they were less likely to forget about it.
      “I do like the concept of the learning plan because … if I didn’t write it down I wouldn’t remember it, so I really do, I really found it useful to write down what topics I needed to know more about. Because it would then give me something to focus on.” (GP202)
    Brings focus to learning
      Learning plans were recognised by some registrars, GPs, supervisors and MEs as a way to focus learning, and were viewed as particularly helpful for some at the start of training. Some RTPs provided structured learning lists for registrars to help guide their learning initially, which was well received by those who struggled to self-direct their learning. Some registrars and GPs found a learning plan useful for exam preparation, and others found it a helpful tool to anchor discussions with their supervisor.
      “… when I started using it in my GPT1 I found it really helpful … it actually keeps a track of my planning.” (GP301)
    Delayed appreciation – value of learning plans not being appreciated until later
      A small number of registrars and GPs reported to appreciate learning plans either later in their training (i.e. GPT3/4) or after they completed their training.
      “… in the last year or so when I was kind of thinking this actually wasn’t a bad idea then I started to be more kind of pragmatic about it and thought it was actually kind of a useful thing.” (GP102)
    Promoting reflection
      A learning plan was identified by some GPs, supervisors and MEs as a good way to encourage reflection for registrars who might not naturally do so. It provided a means for registrars to appreciate their learning deficits and to look back on what had or had not been learned. It was also said to provide insight into how registrars think.
      “The one thing that is helpful is that when the registrar has to make their own learning plan you can see how they think in structuring in time, and so the ones that come with five pages worth of, they need to learn everything about everything, you can quickly identify that the 15 min succinct thinking and planning is going to be their difficulty, and those who turn up with, ‘I don’t know,’ gives you the other end of the spectrum.” (RTP1, Supervisors)
    Helpful for remediation
      One supervisor shared how learning plans were used successfully as a tool for addressing learning deficiencies for registrars in remediation.
      “I have also been involved in remediating a number of registrars who’ve failed exams and when you actually make them produce a learning plan that addresses the active deficiencies that are identified, they actually improve their performance so it’s a useful tool.” (RTP3, Supervisors)
    Encourages independent thinking and self-directed learning
      While some registrars preferred to be provided with an external learning structure, particularly early in their training, others acknowledged the utility of having a self-directed plan.
      “So I find my current learning plan so much more useful because it’s entirely directed by me and it’s focused on what I’m seeing every day.” (RTP2, GPT3&4 s)
    Good idea at a macro level
      The utility and benefit of learning planning was recognised by some registrars as valuable in principle, but appeared to come undone in the delivery. For example, identifying learning needs and thinking about how one might go about addressing them was considered important. But detailing this in a structured learning plan was viewed as counter-productive.
      “I do like what it’s trying to achieve but I don’t know that it quite does it very effectively.” (RTP1, GPT3&4 s)
  Negative perceptions
    Bureaucratic exercise
      Completing a learning plan was overwhelmingly perceived as a bureaucratic exercise across all participant groups. Documentation of learning needs and goals were seen to be primarily for external review, otherwise holding little use or meaning for registrars. It was often considered a “tick box” exercise and was not reported to greatly aid registrar learning. Many registrars reported to complete their learning plan immediately prior to a visit by a medical educator. It was also noted that documenting learning in a learning plan did not guarantee that learning had actually occurred.
      “I only fill the learning planner in to satisfy the requirement that it’s filled in. It doesn’t actually aid my learning.” (RTP3, GPT1&2 s)
    Learning plans unsuitable for some learning styles
      Many participants across all participant groups acknowledged that people had different approaches to learning and planning and expressed the need to allow for individual preference and learning style during training – which may or may not include a formal learning plan.
      “… you’re sort of forcing your learning experience to conform to something that somebody else has kind of put upon you.” (GP102)
    A waste of time and an impediment
      Documenting learning planning in a learning plan was an unpopular process across all participant groups, with many perceiving learning plans being without benefit. It was perceived as a waste of time and unnecessarily time consuming, had little meaning for some, and reflected an artificially structured process that made planning more complicated than it needed to be.
      “I don’t like doing the learning planner because it’s a waste of my time to try and dig out what I need to know, put it onto the system and then learn from that.” (RTP3, GPT1&2 s)
    Learning planning as a verb, not a noun
      This was expressed by some MEs who considered learning planning as a dynamic process that was not amenable to static documentation in a formal learning plan.
      “It should be a verb not a noun, and the trouble is it’s talked about as a noun, but it’s actually a verb, it’s a process, it’s an action thing, but it gets talked about as a noun, like something that you can hold or look at. And that’s where it gets all mixed up because we get so focused on the noun and we don’t concentrate on the verb, which is how do you actually learn? And what’s your process for learning? That’s where the problem is.” (RTP2, MEs)
      Emphasis was placed on the dynamic nature of learning planning, which meant a formal learning plan was in some ways inadequate to capture a registrar’s learning. It was also argued that experienced GPs themselves do not use learning plans.
      “It’s mandated so my job as a supervisor is to say you’ve got to fill that sucker in but in my own occasion I’ve been doing exams for 35 years, I don’t go and write a formal learning plan.” (RTP3, Supervisors)
      The idea of a learning plan being a verbal plan, instead of written, was also raised by supervisors.
      “I agree that what we’re talking about this sort of floating learning plan that you work out on the run and update verbally, is probably much more a useful thing.” (RTP1, Supervisors)
    Perceived lack of evidence and therefore lack of ‘buy in’
      Many participants across all participant groups questioned what evidence there was for the effectiveness of learning plans.
      “Some ivory tower educational thing, probably wasn’t in medicine, it was probably…It was someone in that educational ivory tower or sitting in Canberra [Australia’s Capital City] who decided that’s a good idea.” (RTP3, Supervisors)
      There was variation across supervisors and MEs in terms of buy-in; some believed them to be effective and helpful, others were adamantly opposed, and others stood somewhere in the middle. Some questioned their use, because they themselves had never completed a learning plan. Some MEs continued to support and encourage registrar use of formal learning plans, despite their lack of buy-in, simply because they were a requirement of training.
      “So we try to tow the party line without really believing in it ourselves.” (RTP3, MEs)
    Unsuitable for adult learners
      Given registrars were adult learners with many years of successful study behind them, mandated formal learning plans were considered by many registrars, GPs, supervisors and MEs as condescending and even insulting.
      “I feel like it’s an insult to our intelligence; like you’ve gone through bloody 20 years of study plus and you know, we know how to learn, we’re not dumb.” (RTP3, GPT1&2 s)
      “It’s just such a stupid concept for high achieving adults, which is what we’re dealing with, it’s just the most ridiculous idea.” (RTP2, MEs)
    Does not work in the context of work-based learning
      Formal learning plans were reported to not necessarily reflect the natural way learning occurs in busy general practice clinics particularly the ‘just in time’ learning that occurs in the workplace.
      “There is a whole lot of just in time stuff that I think kind of can really go missing if everything turns into a formal kind of process.” (RTP1, Supervisors)
      Some also expressed difficulty knowing when some learning needs had been achieved.
      “… one of the questions on … their sample learning planner was, ‘how will I know when I’ve achieved that?’ And that’s a pretty difficult question to answer … you know, be able to do a good pap smear, well how do you know when you, when you’ve mastered that, like when the patient doesn’t jump up off the bed and run out flinging the speculum back at you?!” (GP102)
    Exposing competency gaps
      Documenting learning needs in a learning plan exposes a registrar’s competency gaps, and for some, this was a cause for embarrassment. This was expressed across all participant groups. It was confronting for those who perceived their learning needs as weaknesses, and were reluctant to reveal this to others.
      “No one wants to write it down. No one wants to write down the weak spots.” (RTP3, GPT3&4 s)

## Discussion

Our study describes the use and perception of formal learning plans by GP registrars, recently trained GPs, supervisors and MEs across three RTPs in Australia. The strength of our study lies in the breadth of participants sampled, the broad range of Australian GP training contexts that they came from and the range in types of learning plan data collected.

We found learning plans were not well accepted, nor utilised, by most learners, supervisors and educators across the three RTPs. While the majority of registrars were technically meeting training requirements by filling in a learning plan each term, they commonly did not use the tool as intended. For online learning plans, learning needs were often entered and ticked off as completed within the same day, suggesting learning plans were used predominantly as a log of activities instead of an active planning tool. Some admitted to forgetting about learning needs that were initially written down and fabricating parts of their learning plan to satisfy requirements, which has also been reported in the literature [6, 7]. This suggests an external driver of learning plan use, rather than genuine engagement in the use of the learning plan. RTP learning plans evaluated as part of this study rarely made a meaningful contribution to registrar learning planning [23].

Lack of learner and educator buy-in concurs with previous research reporting the low rate of acceptability of learning plans in medicine [10, 12] and in general practice [9, 11]. Learning plans were also perceived by many as rigid bureaucratic impositions that did not usually match the learners’ preferred method of planning their learning [12, 24, 25].

Further, we found learning plans were not well accepted by registrars for reasons such as lack of applicability to workplace learning, poor match to individual learning preferences, lack of time, and difficulty establishing or working with meaningful discrete goals; all of which detracted from the perception of learning plans as an effective learning tool [12].

### Supervisor use and perceptions of learning plans

Numerous studies point to the important role of a supervisor in the successful use and execution of a learner’s learning plan [24, 26–28]. Similarly, we found that registrars were more likely to use their RTP learning plan if their supervisor was supportive and took an active interest in it. Learning plans also brought a focus to learning in early phases of training, and provided structure to registrar-supervisor meetings [3]. However, the majority of supervisors in our study did not actively engage in or encourage learning plan use with registrars; nor did they perceive learning plans to be a useful tool. Retrospective learning plan usage data from one RTP indicated that supervisors rarely accessed their registrars’ online learning plans, despite the software being set up in a manner that could be used by them. Learning planning was often verbal and informal. A learning plan (when used) was reported to provide focus to teaching sessions, but the impetus was on the registrar to initiate its use. Whilst registrar engagement was reportedly higher when a supervisor was more engaged with the learning plan, our qualitative data demonstrate that this did not guarantee learning had taken place.

### The place of learning plans in GP vocational training

GP vocational training occurs in environments whose prime purpose is service delivery associated with patient care; the learning and reflection that occurs is multi-faceted and complex [29]. Whilst the impetus for introducing learning plans and portfolios was to promote a self-directed learning process and enable individuals to monitor their progress [5], it is perhaps counterproductive to mandate their use. Mandating the use of formal learning plans in this context will struggle to be entirely accepted.

Low acceptability of learning plans among learners and their educators in our study and others [12, 30] does not necessarily mean this tool is ineffective for facilitating learning. Our data suggested that some registrars did find their learning plan a useful tool to direct their learning [12] which seemed to align with their self-reported, individually preferred learning style. It is also possible that a formal learning plan promotes effective learning, even if the users do not like it.

Formal learning plans reflect an individualistic approach to learning where an individual’s characteristics (e.g. self-confidence in self-directed learning) have been found to be more influential than program characteristics (e.g. program level support for learning plans) for achieving learning goals [10]. If learning plans are to achieve a higher rate of acceptability among registrars, it is possible that one way of doing this is helping registrars develop confidence with self-directed learning [10] and to be clear about why they are important and valuable. Our study demonstrates that it’s important to communicate the evidence for the utility of learning plans to establish buy-in.

However, given the low acceptability of formal learning plans by most learners and educators in our study, and in particular, the view that learning plans were condescending and even insulting, the place of learning plans as a mandated tool in GP vocational training needs to be seriously questioned. Rather than mandating the production of a formal learning plan, it may be more justifiable to mandate evidence of learning planning which may be demonstrated in more ways than submitting a formal learning plan.

### Theories of learning

Learning planning and learning plan use have traditionally been understood from the perspective of adult learning theory. From this perspective learning serves the learner, and the learner holds primary responsibility for the learning. It also suggests that learning is a linear process where a need is identified, a learning activity determined and undertaken and learning reflected on. Findings from our study suggest that registrar learning may be better understood as a social process. Social learning theories hold that learning is as much the concern of the communities in which learning occurs as it is of the learner. It is also about material artefacts and other social affordances. Most learning is implicit rather than explicit [2]. Taking this approach then shifts how we regard the process of learning planning from the traditional perspective of registrars as a relatively independent agent of their own learning, learning in a linear way, to one in which representatives of the broader communities of education, work practice and patients play important roles and where learning is much more organic.

As demonstrated by the findings of our study, a registrar’s experience of learning planning and writing a learning plan are varied. It is impacted on by the individual registrar, others in their learning community (e.g. supervisors, MEs, peers, patients), material artefacts (e.g. computers, post-it notes, books), and social structures such as the professional colleges. As such, a communities of practice [18] approach has utility, as does a socio-material approach that reclaim materials and materiality in social life (i.e. considering the resources, notebooks and learning plans as integral to the experience of learning planning and not just as background tools) [19].

### Study strengths and limitations

Collection of de-identified retrospective data on registrars’ learning plan use offered a clear picture of how learning plans were used without the bias of self-report. We were unable to collect comparable data from one of the RTPs due to differences in the learning plans used, and the records kept of learning plan use. This was a foreseen limitation to this component of the study, which was outweighed by the value of qualitatively exploring and evaluating the three different RTP approaches to learning plans.

Qualitative data complimented the usage data and provided deeper insight. Inclusion of multiple RTPs across Australia and with different participant groups enabled us to address our research questions from multiple perspectives and contexts. This revealed strong agreement across participant groups and RTPs, providing a consistent picture of how learning plans are perceived and used, irrespective of the tool and approach taken by the RTP. Together with what we know from the literature regarding the low acceptability of learning plans among learners [12, 30] it is likely that our findings provide some generalisability to other registrars engaged in general practice vocational training in Australia.

## Conclusions

Learning plans are an integral and mandatory part of GP vocational training in Australia and form part of the credentialing process to assess registrar competence. Whilst structured learning plans had the capacity to be useful for a minority of learners, for many this was not the case. This study provides creditable evidence that learning plans are broadly considered by users to be a bureaucratic impediment with little value as a learning tool. Learning plans were perceived to provide little benefit to registrars in their journey to becoming a competent GP. The issue that needs to be addressed is whether current individual registrar learning planning practices are effective and efficient in enabling registrars to become confident competent practitioners. Findings from this study suggest it is more important to support registrars in *planning* their learning than to enforce documentation of this process in a learning plan. If learning planning is to be an assessed competence, methods of assessment other than the submission of a formal learning plan should be explored.

## Additional files

Additional file 1: Learning plan types across the three RTPs. Provides detail about the types of learning plans used by the three RTPs, as well as what the RTP required of registrars when completing their learning plan. (PDF 292 kb) Additional file 2: Focus group and interview schedules. Provides the questions used during focus groups for GP supervisors, medical educators and registrars, and interviews with recently trained GPs. (PDF 277 kb) Additional file 3: Learning plan use at RTP 2 (paper-based). Provides additional data about RTP 2 based on 38 registrar exit-interviews during 2014 when registrars were asked about utility of the learning plan. Includes exit interview registrar characteristics, number of registrars reporting the learning planner as useful/not useful, and qualitative reflections on the learning planner. (PDF 227 kb)

## Abbreviations

AGPT: Australian General Practice Training
AMG: Australian Medical Graduate
GP: General practice
IMG: International Medical Graduate
ME: Medical Educator
NZMG: New Zealand Medical Graduate
RTP: Regional Training Provider
