# Supplementary material for: Utility of learning plans in general practice vocational training: a mixed-methods national study of registrar, supervisor, and educator perspectives
Source: BMC Med Educ. 2016 Aug 19;16:211. doi: 10.1186/s12909-016-0736-8 (PMC4992211; doi:10.1186/s12909-016-0736-8)
Supplement: Additional file 1: — Learning plan types across the three RTPs. Provides detail about the types of learning plans used by the three RTPs, as well as what the RTP required of registrars when completing their learning plan. (PDF 292 kb) [file 12909_2016_736_MOESM1_ESM.pdf]

Additional file 1

Learning plan types across the three RTPs

RTP 1

RTP 1 had recently transitioned from an electronic, static document to an interactive online learning plan

Electronic, static document

This learning plan was an electronic document that registrars were required to complete and have signed off by a supervisor and training advisor each semester. It comprised six sections: academic achievement; communication/consultation; cultural safety; knowledge; professionalism and teamwork. Registrars were required to answer a set of questions within each section:

- What is my objective?
- Why do I need to learn this?
- What resources will I need?
- Date/record of activity
- How will I know when I've reached my objective?
- Possible future learning objectives?

Registrars had the option of completing these sections electronically, or printing out the documents and completing them by hand.

The learning plan was designed to encourage registrar reflection on areas of their practice that needed further development, gaps in knowledge, and development of a specific plan to address these needs. GP supervisors and MEs were highlighted as playing an important role in supporting the registrar to formulate a learning plan, and progress through the learning needs documented in the plan.

Interactive online learning plan

In January 2015, RTP 1 launched a new online educational platform. The registrar learning plan was housed within this platform. In its first roll-out, GPT1s and GPT2s were provided with a fixed learning plan within the program. This learning plan provided a prescribed list of weekly learning topics and resources for registrars to access on each topic. The learning plan provided a central text communication function that enabled registrars and supervisors to post messages to each other to discuss the learning items.

RTP 1 registrars were required to complete the learning plan each term.

RTP 2

This training provider utilised a hard copy learning plan folder designed to provide registrars with a resource that they could use for the life of their training. This was given to them at orientation. The folder contained information about learning planning, lists of available educational support and resources, and partitioned sections with learning plans, patient logs and other materials relevant to each term. For example, the GPT1 section provided registrars with a suggested timeline for completion of the learning planner, two self-assessment tools ('Domains of GP' and 'Procedural Skills'), a patient log, learning plan, and a learning and teaching record.

The patient log required registrars to keep a record of at least 15 consecutive patients each term. Details to be recorded by registrars included: patient age, gender and reason for encounter; issues raised for the registrar; and learning needs identified from the encounter.

The learning plan was a simple one page document that required registrars to identify a set of learning objectives, the resources and methods they may use to meet those objectives, and their methods for measuring progress. Registrars were asked to have the learning plan signed off by a supervisor or training advisor (TA) and make a copy to submit to the RTP. In practice, submission of learning plan forms was not a strict requirement and was not always followed up by the RTP. However, registrars were engaged in conversations about their learning planning with their medical educators.

In the folder's written materials, registrars were encouraged to complete and bring their learning plan to each GPT1 and GPT2 workshop and meetings with their educators, including external clinical training visits and training appraisals. Whilst encouraged, it was not a strict requirement.

Registrars were also provided with suggested timelines for completion of their learning plan and other tasks.

RTP 3

This training provider required registrars to maintain a learning plan for the duration of the general practice training program. They provided registrars with access to a web-based learning planner. This electronic learning planner was designed to encourage deliberative learning by offering:

- An online planning tool that could be accessed and used at any time and location to document learning needs, activities, outcomes and reflections;
- Accessibility for registrars, supervisors and educators (potentially facilitating discussion and collaborative identification of learning needs);
- Learning opportunities tools including listings of facility opportunities, upcoming events, RTP monthly themes and mandated activities that can be directly entered to the learning plan.
- Learning needs tools, including common presentations, RACGP and ACRRM curriculum and a procedural skills log that can also be used to directly enter items to the learning plan.

Registrars were introduced to the learning planner at their GPT1 introductory workshop. The learning planner was then discussed one to two months later at the first training advisor visit. Registrars were also encouraged to discuss and commence their learning plan with their supervisor within four weeks of commencing their placement.

The learning plan was intended to be an active document, informed by regular discussions between a registrar and his or her supervisor. The aspiration was for the learning plan to be actively read and 'acknowledged' a number of times during a semester by a supervisor. The online platform used had a 'Viewing history' function where supervisors and medical educators could indicate that the learning plan had been viewed. Ideally, the learning plan was viewed and 'signed-off' at the end of each training semester by the registrar's supervisor and/or Training Advisor (TA). Registrars were each visited once per semester by a TA, who checked on the registrar's learning planning and encouraged use of the learning plan as needed. The TA may have (but did not always) record details of the registrar's learning plan use at each visit. Learning plan completion was, however, consistently verified at the completion of a registrar's training.

The online learning planner had a 'semester focus' that encouraged registrars and their supervisor to develop a new plan at the beginning of each semester. There was a semester migration tool that enabled registrars to carry-over any items that were not completed in a previous semester.

Registrars were entitled to use means other than the online learning planner to document their learning, provided that the RTP requirements were fulfilled. The registrar needed to ensure that a learning plan could be submitted to the RTP, either as a hard copy or an electronic document.
