# Supplementary material for: Utility of learning plans in general practice vocational training: a mixed-methods national study of registrar, supervisor, and educator perspectives
Source: BMC Med Educ. 2016 Aug 19;16:211. doi: 10.1186/s12909-016-0736-8 (PMC4992211; doi:10.1186/s12909-016-0736-8)
Supplement: Additional file 2: — Focus group and interview schedules. Provides the questions used during focus groups for GP supervisors, medical educators and registrars, and interviews with recently trained GPs. (PDF 277 kb) [file 12909_2016_736_MOESM2_ESM.pdf]

Additional file 2

FOCUS GROUP SCHEDULE: REGISTRARS

INTRODUCTION

Identifying learning needs

1. Can you tell us how you become aware of things you need to learn?

2. Do you prioritise or favour some learning needs over others?

Planning to address a learning need

3. Once you've identified a learning need - what happens next?

Formal learning planning

4. We'd like to explore your thoughts and experiences of structured learning plans. Can you tell us about the learning plan(s) provided by your RTP?

5. Do you use the learning planner provided by your RTP?

6. What would encourage you to use, or improve your use of, learning plans?

7. What do you think about formal learning planning as a mandated process?

Meeting and reflecting on learning needs

8. Do you spend time reflecting on your learning? (Learning needs, new knowledge)

Other questions

9. What have been your most powerful learning experiences (examples?).

CONCLUSION

FOCUS GROUP SCHEDULE: MEDICAL EDUCATORS

INTRODUCTION

Identifying and addressing registrar learning needs

1. Can you tell us about your role as medical educators, and how your role intersects with registrars and GP supervisors?

2. Thinking about registrars and their learning…. In your experience, are registrars capable of identifying their learning needs?

3. What is your role, as an ME, in assisting registrars to identify and address these weaknesses/gaps or areas of learning need?

4. Do you think registrars tend towards learning activities that they already know about and feel comfortable with?

Formal learning planning

We'd like to explore your thoughts and experiences of structured learning plans.

5. Can you tell us about the LP that your registrars are asked to complete?

6. What is your role, as an ME, in registrars' learning plan completion?

7. Do you receive any kind of orientation or instruction from the RTP about the learning plan, e.g., how it should be used by registrars, your involvement.

8. On the whole, what do you think of the use of LPs in registrar training?

Improving the learning planning process

9. How could the learning plan model offered via your RTP better meet the needs of registrars and supervisors?

Broader questions about facilitating registrar learning

10. What do you think are the most powerful learning experiences for registrars?

11. What makes for effective learning?

12. What makes for effective learning planning?

13. What place do formal learning plans have in this process?

CONCLUSION

FOCUS GROUP SCHEDULE: GP SUPERVISORS

INTRODUCTION

Identifying and addressing registrar learning needs

1. Can you tell us about your role as GP supervisor? (Broad opening question)

2. Thinking about deliberate learning by registrars, what do you see your role as in assisting them to identify and address their learning needs? Can you tell us about this?

3. Do you or your registrar document what you have just described?

4. Are there important areas that registrars tend to miss? Explore reasons why (e.g., they feel uncomfortable about a particular area/avoidance, simple ignorance).

Formal learning planning

We'd like to explore your thoughts and experiences of structured learning plans.

5. Can you tell us about the learning plan that your registrars are asked to complete?

6. What is your role in registrars' learning plan completion?

7. On the whole, what do you think of the use of LPs in registrar training?

Improving the learning planning process

8. How could the learning plan model offered via your RTP better meet the needs of registrars and supervisors?

CONCLUSION

INTERVIEW SCHEDULE: RECENTLY TRAINED GPs

INTRODUCTION

Use and experience of LPs

1. Can you tell me about the LP that you were asked to complete during training?

2. Did you use this, or another form of LP, during your registrar training?

    2a. If yes…. what was your experience of using the LP?

    2.b. If no….. could you tell me about why you didn't use a LP?

3. Can you tell me a bit more about your process of learning during your training?

4. What level of involvement did your GP supervisor have in your learning planning?

5. What level of involvement did your RTP have in your learning planning?

Improving the learning planning process

6. How could the learning planner offered via your RTP better meet the needs of registrars during their training?

Knowledge and skill gaps

7. Now that you've completed your training, are you aware of particular gaps in your knowledge & skills as a GP? Could you tell me about these?

CONCLUSION
