# Supplementary material for: Utility of learning plans in general practice vocational training: a mixed-methods national study of registrar, supervisor, and educator perspectives
Source: BMC Med Educ. 2016 Aug 19;16:211. doi: 10.1186/s12909-016-0736-8 (PMC4992211; doi:10.1186/s12909-016-0736-8)
Supplement: Additional file 3: — Learning plan use at RTP 2 (paper-based). Provides additional data about RTP 2 based on 38 registrar exit-interviews during 2014 when registrars were asked about utility of the learning plan. Includes exit interview registrar characteristics, number of registrars reporting the learning planner as useful/not useful, and qualitative reflections on the learning planner. (PDF 227 kb) [file 12909_2016_736_MOESM3_ESM.pdf]

Additional file 3

Learning plan use at RTP 2 (paper-based)

Given the learning planner at this RTP was paper-based, unfortunately no data pertaining to learning plan use and completion were available. However, we were able to examine end-of-training interview data relating to registrars' reflections on two questions: "Is the learning plan useful?" and "How could it be improved?".

Registrar reflections were extracted from 38 registrar exit interviews undertaken in 2014 and the first half of 2015 (Table 1).

Table 1. Exit interview registrar characteristics.

Characteristic	n (%)
Gender	
Female	24 (63%)
Male	14 (37%)
Cohort	
2008	3 (8%)
2009	0 (0%)
2010	2 (5%)
2011	8 (21%)
2012	16 (42%)
2013	9 (24%)
Graduate	
AMG	28 (74%)
IMG	10 (26%)

AMG = Australian Medical Graduate
IMG = International medical Graduate

As displayed in Figure 1, over half of the registrars did not find the learning plan useful. However, just over a third of registrars (37%) indicated that they did find the learning plan useful. Chi square tests of independence were performed to examine whether there were any significant correlations between registrar characteristics (gender, cohort and IMG/AMG status) and perceived usefulness of learning plans. No significant relationships were identified ($p>0.05$).

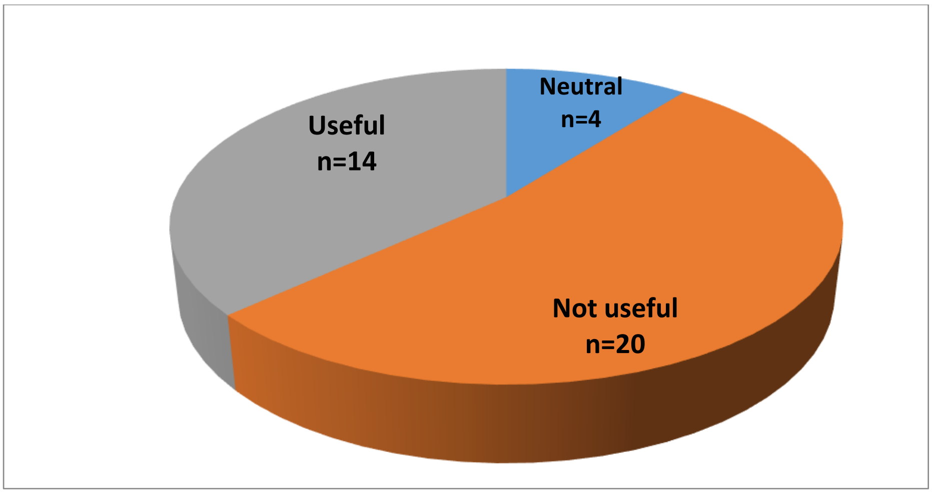

Figure 1. Registrar reflections on whether they found the learning planner useful.

Registrars' reflections on the learning planner were reviewed and broken down into beneficial aspects and negative reflections (Table 2 & 3). **Many registrars indicated that they did not use the learning planner, and nor did they find it useful.** However some registrars indicated that they found the tool helpful for some aspects of their learning, and more broadly, it encouraged the notion of learning planning.

Table 2. Beneficial aspects: RTP 2 registrar reflections on their learning planner.

Aspects of the learning planner that registrars found beneficial

- Several registrars indicated that the learning plan assisted them to identify their areas of weakness and to address gaps in their knowledge.
- While some registrars did not necessarily find the learning plan tool itself helpful, several indicated that it encouraged the broader notion of learning planning and offered them some direction and focus.
- The tool was more likely to be regarded as useful at the beginning of training, rather than later in training.
- Some found the tool useful for exam preparation, particularly the topic outline.
- The tool can be useful to discuss learning needs and activities with a supervisor.
- Small numbers of registrars identified specific features or ways in which the learning plan tool was helpful: the patient log; study schedules; and the folder itself was useful for keeping documentation together.

Table 3. Negative reflections: RTP 2 registrar reflections on their learning planner.

Negative reflections on the learning planner

- Generalised negative feedback: “Waste of time”, "Tedious and useless", “Did not use it all”, “No benefit in its current state”, “Did not look at it once! Overwhelming”, “Didn’t get anything out of it”.
- Lack of supervisor follow-up and/or interest: “[I] worked out half way through that no one was going to check it regularly so didn't write in it so often”, “Not at all. One reason - supervisors don't support the use of them”.
- Some registrars found that it didn’t suit their style of learning and/or they had their own approach to learning planning.
- Some regarded it as extra paperwork, did not see the benefit in documenting their learning, and did not want to spend time completing it without benefit.
